# Economic Theory and Self-Reported Measures of Presenteeism in Musculoskeletal Disease

**DOI:** 10.1007/s11926-016-0600-1

**Published:** 2016-07-11

**Authors:** Cheryl Jones, Katherine Payne, Brenda Gannon, Suzanne Verstappen

**Affiliations:** Manchester Centre for Health Economics, The University of Manchester, 4th Floor, Jean McFarlane Building, Oxford Road, M13 9PL Manchester, UK; Arthritis Research UK/MRC Centre for Musculoskeletal Health and Work, University of Southampton, Southampton, UK; Arthritis Research UK Centre for Epidemiology, Centre for Musculoskeletal Research, Institute of Inflammation and Repair, The University of Manchester, Manchester Academic Health Science Centre, Stopford Building, Oxford Road, M13 9PT Manchester, UK; NIHR Manchester Musculoskeletal Biomedical Research Unit, Central Manchester NHS Foundation Trust, Manchester Academic Health Sciences Centre, Manchester, UK

**Keywords:** Systematic review, Self-report presenteeism instruments, Musculoskeletal diseases, Economic theory

## Abstract

This study had two objectives: to describe the historical development of self-reported presenteeism instruments that can be used to identify and measure presenteeism as a result of musculoskeletal disease (MSD) and to identify if, and how many of these, presenteeism instruments are underpinned by economic theory. Systematic search methods were applied to identify self-report instruments used to quantify presenteeism caused by MSD. A total of 24 self-reported presenteeism instruments were identified; 24 were designed for use in general health, and 1 was specifically designed for use in rheumatoid arthritis. One generic self-reported presenteeism instrument was explicitly reported to be underpinned by economic theory. Overtime, self-reported presenteeism instruments have become more differentiated and complex by incorporating many different contextual factors that may impact levels of presenteeism. Researchers are encouraged to further develop presenteeism instruments that are underpinned by relevant economic theory and informed by robust empirical research.

## Introduction

Since 1990, the global burden of musculoskeletal diseases (MSDs), including chronic rheumatological conditions, as measured by disability-adjusted life years (DALYs), has been shown to increase dramatically [1]. For many chronic rheumatological conditions, such as rheumatoid arthritis (RA), psoriatic arthritis (PsA) and ankylosing spondylitis (AS), disease onset can occur at any age; however, peak incidence rates have been found to occur between the ages of 40 and 65 years [2]. Previous reviews have estimated that within 13 years after onset RA, the probability of becoming work-disabled is 50 % [3, 4]. Similarly, after 5 years of the onset of AS, up to 13 % of adults are likely to lose their jobs [5]. The reduction in health status in people with these diseases will not only affect their daily functioning, and cause early mortality, but may also have a major impact on their productivity at work (productivity loss). In the last two decades, there have been some advances in the treatment of MSD, but these strategies are not curative and many people still experience productivity loss [6–8].

Productivity can be viewed, within the context of the employment environment, as a measure of technical efficiency that examines how inputs, such as labour and capital (technology), are used to produce outputs of sufficient volume and quality [9]. The productive rate of any individual may be affected by a variety of factors such as job demands, levels of support, working hours, job satisfaction and, perhaps most importantly, poor health. The impact of a reduction in health status has been directly linked to productivity loss through absenteeism and presenteeism. Absenteeism refers to the time (hours, days, weeks) spent *away* from work because of illness [10–13], and presenteeism refers to the reduction in ‘working performance whilst at work due to ill health’ [14].

It is relatively easy to objectively quantify absenteeism using simple counts of days away from work. Quantifying the impact of presenteeism is much more challenging, involving two stages: (1) identifying and measuring the volume of unproductive time and (2) valuing the impact of that unproductive time. The lack of available objective measures that can be used to identify and measure presenteeism has led to the development of instruments that rely on self-reports from the individual affected by an adverse health condition, such as a MSD.

A number of reviews have systematically identified a number of instruments, both general health and disease-specific, that are available to self-report presenteeism. Some of these systematic reviews have focused on how to identify and measure presenteeism and found that the available instruments differed extensively and lead to vastly different estimations of the volume of presenteeism [11, 15, 16]. In contrast, the methods used to value the impact of presenteeism has largely focused on using two, similar, methods that centre on using cost as the unit of measurement: the human capital approach (HCA) and the friction cost approach (FCA) [17]. The HCA and FCA value the amount of productivity loss by multiplying the amount of time an individual is unproductive during a working week by the wage rate. The two methods differ in terms of the perspective they take. The HCA calculates the cost of lost productivity from the perspective of the patient/employee. Therefore, the cost of lost productivity continues until that individual employee/patient has found another job. The FCA takes an employer perspective and calculates the cost of lost productivity based on the amount of time it takes to replace the sick employee; this period of time is known as the ‘friction’ period. Once the sick employee has been replaced, the FCA assumes that initial production levels are restored [17]. The HCA and the FCA are grounded in economic theory that assumes that productivity is equal to the market wage which represents the marginal revenue product of labour of an employee working for an employer in the context of a perfectly competitive market [18]. Two studies by Pauly et al. [19] and Zhang et al. [12] criticise the economic theories that are used to underpin methods that value the cost of lost productivity. Pauly et al. suggested that the allowances for sick days and protection against fluctuating wages will mean that employees will accept a wage rate that is lower than the value of the marginal productivity of the worker. Therefore, the value of productivity loss will exceed the value of the wage because the wage is lower than marginal productivity. Similarly, Zhang et al. argued that the cost of productivity loss will exceed the value of the wage rate if a job involved team-based work, unavailability of substitutes, and produces highly time-sensitive output.

It is important to understand the theoretical underpinning of an approach to quantify the impact of a subjective construct, such as presenteeism, to enable the development of a robust approach to its identification, measurement and valuation. Economic theories provide a common framework from which to develop methods that can be used to identify and measure the impact of presenteeism. To our knowledge, it is currently unknown which, if any, self-reported presenteeism instrument used to identify and measure presenteeism is underpinned by economic theory. This study had two objectives: (1) to describe the historical development of self-reported instruments that can be used to identify and measure presenteeism as a result of a MSD and (2) to identify if, and how many of these, self-reported presenteeism instruments are underpinned by economic theory.

## Methods

A systematic review was carried out to identify all published studies that describe the development of self-reported presenteeism instruments that can be used in MSD. The search was run up until November 2015. The systematic review was conducted, in line with advice and guidelines published by the Centre for Reviews and Dissemination (CRD) [20] and followed the Preferred Reporting Items for Systematic Reviews and Meta-Analyses (PRISMA) checklist.

## Search Strategy

The search for relevant studies involved updating a recent systematic search conducted by Ospina et al. in 2012 [21•]. Ospina et al. conducted a systematic search that identified all general health and disease-specific presenteeism instruments. The electronic search strategies used by Ospina et al. (see Appendix) were retrieved and re-ran in eight electronic databases including Medline (1946 to September week 3 2015), Embase (1980 to week 40 2015), Cochrane Central Register of Controlled Trials (CENTRAL) (August 2015), PsychINFO (1806 to September week 4 2015), Web of Science (1900 to November 6, 2015), CINAHL (1937 to November 6, 2015), Business Source Complete (1886 to November 6, 2015) and ABI inform (1970 to November 6, 2015). The electronic search strategies comprised of the specific names of presenteeism instruments, such as the ‘Endicott work productivity scale’ and more generic terms such as ‘productivity’ and ‘presenteeism’. The new search was constrained to run between 1st January, 2012 to 6th November, 2015.

## Study Selection Process

All titles and abstracts identified were double screened for inclusion by two independent reviewers (CJ and either KP, BG or SV) and accepted if the study met the inclusion criteria specified in Table 1.

**Table 1:** Inclusion criteria

	Inclusion criteria	Exclusion criteria
Study type	Development of method that quantifies presenteeism	Studies that apply the developed method, for example, in economic evaluations
		Studies that test methods of presenteeism in terms of their psychometric properties *and* do not discuss the development of the instrument
Focus	Methods developed for assessing health-related presenteeism	Methods developed for assessing other forms of productivity loss, e.g. shirking
	Methods developed for assessing generic health or musculoskeletal conditions	Methods developed that focus on disease-specific areas except musculoskeletal conditions, e.g. mental health
	Original development of presenteeism methods	Adaptations of methods for use in other countries, e.g. WLQ-J
		Adaptations of methods for use in specific disease areas if the original was developed for general health
Publication type	English language	Foreign languages

## Data Extraction and Synthesis

One reviewer (CJ) extracted the data from each study using a bespoke data collection form to extract author, year and country that the study was completed; name of the presenteeism instrument; aims of the instrument; whether the instrument also measured absenteeism; the structure of the instrument; recall period used; whether estimations of presenteeism using that specific instrument could be converted into monetary values using the HCA or FCA; and clear reporting of the economic theories used to underpin the presenteeism instrument developed. The results were tabulated and summarised as part of a narrative synthesis.

## Results

**Fig. 1:**
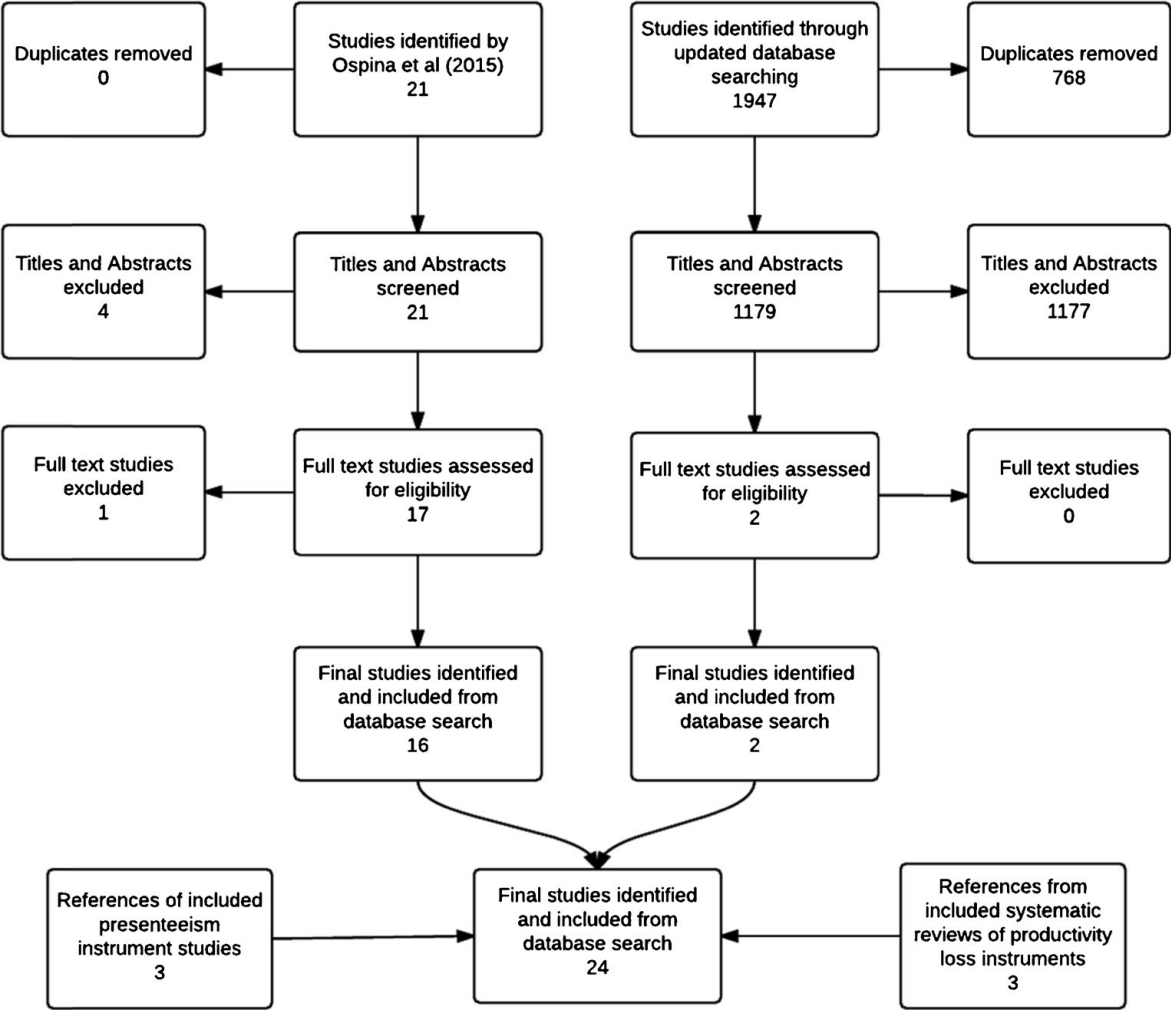
Flow diagram of study selection process

In total, 24 studies that described the development of presenteeism instruments for use in MSD were identified. Of these, the Work Productivity Survey for Rheumatoid Arthritis (WPS-RA) [22] was the only one designed to specifically measure presenteeism associated with a MSD (RA). The remaining 24 presenteeism instruments were developed for use across a wide range of health conditions, including MSD. Figure 1 illustrates the identification and inclusion of relevant studies. A summary of the identified presenteeism instruments are presented in Table 2.

**Table 2:** Summary of presenteeism instruments

Author, year country^a^	Name	Aims	General health or MSD	Absenteeism also measured?	Structure of instrument	Recall period	Monetise productivity loss?	Economic theory
Jette et al., 1986, USA [23]	The Functional Status Questionnaire/Work Performance Scale (WPS)	Screen disability and monitor clinically meaningful change in function	General	Yes	Four domain scale: 1. Physical functioning 2. Psychological functioning 3. Social/role functioning 4. Six single-item questions Respondents answer a set of statements and assign a grade, for example, 1 = usually did not complete and 4 = no difficulty.	1 month	Not reported	No
Reilly et al., 1993, USA [24]	Work Productivity and Activity Impairment Instrument (WPAI)	To measure the effect of general health and symptom severity on work productivity and regular activities. The WPAI uses function related end-points to allow a measure of the economic impact of relative differences of therapeutic interventions	General	Yes	Questionnaire asks for: 1. Number of days and hours missed from work 2. Days and hours worked 3. Number of days work was difficult 4. Extent to which poor health was attributable to work loss 5. Parallel set of questions (1 to 4) about regular activities of unpaid work Respondent asked to rate their own working performance. Overall work productivity calculated as a %.	7 days	HCA	No
Van Roijen et al., 1996, The Netherlands [25]	Health and Labour Questionnaire (HLQ)	Collect data on relationship between illness, treatment and work performance	General	Yes	Four modules: 1. Absenteesim (paid work) 2. Reduced productivity at work (paid) 3) Unpaid work 4) Impediments to paid and unpaid labour Absenteeism: Respondents asked to state whether they performed paid work or not during past 2 weeks. If large sample mean number of work days lost can be calculated by multiplying by 26 Reduced productivity: 7 items. Respondents asked to rate 1 = never to 4 = always. Score equals the sum of all items. Unpaid production: Respondents asked how many hours spent doing unpaid work. These hours are compared to those of the general population or control group. The mean annual hours of unpaid production calculated by multiplying by 26 Impediments to paid and unpaid labour: Respondents asked to state level of impediment experienced whilst performing the job: 0 = no impediment and 3 = a lot of impediment	2 weeks	Not reported	No
Endicott et al., 1997, USA [26]	Endicott Work Productivity Scale (EWPS)	To assess the extent to which a health condition affects the individual’s ability to function at work	General	Yes	25-item questionnaire. Domains: 1. Employment status including self-employed 2. Absenteeism 3. Presenteeism Respondents are asked to rate their performance using a five-point scale: 0 = never and 4 = almost always	1 week	Not reported	No
Kopec and Esdaile, 1998, Canada [27]	Occupational Role Performance (ORQ)	Develop a back pain instrument to measure individual’s ability to perform job	General	Not clear	16 items grouped. Six domains: 1. Amount of time working 2. Productivity/efficiency 3. Quality of work 4. Job satisfaction 5. Job security 6. Relations with co-workers Respondents were asked to choose the response they most associated with; no, a little, somewhat and a lot. Overall score calculated as a sum of item scores and converted to a 0 to 100 range.	2 weeks	Not reported	No
Brouwer, Koopmanschap and Rutten, 1999, The Netherlands [28]	The Quality and Quantity Method (QQ)	Measure the quality and quantity of work performed	General	No	Two domains: 1. Quantity of work 2. Quality of work Respondents asked to rate the quantity and quality of work completed using a scale from 1 to 10.	Last working day	HCA	No
Burton et al., 1999, USA [29]	Work Productivity Index (WPI)	To measure decreased productivity associated with health condition by using an objective measure of productivity	General	Yes	Two domains: 1. Time away from job 2. Time lost to maintain the productivity standard Threshold of productivity established: employee score over 0.5 indicates meeting standard productivity = 0 h lost. Scores less than 0.5 evaluated as a proportion of 0.5 and subtracted from 100 %.	1 week	HCA	No
Amick, et al., 2000 USA [30]	Work Role Functioning Questionnaire (WRFQ)	Through use of literature review, the aim to was discuss advantages and disadvantages of current instruments and to develop the WRFQ	General	Not clear	Five modules: 1. Work scheduling 2. Physical demands of jobs 3. Mental demands of jobs 4. Social demands 5. Output demands Respondents asked to state the amount of time they have had difficulty meeting the demands of their work. 100 % all the time, 50 % half of the time and 0 % none	4 weeks	Not reported	No
Lerner et al., 2001, USA [31]	The Work Limitations Questionnaire (WLQ)	To measure on-the-job impact of chronic conditions and treatment on work productivity	General	Yes	25-item questionnaire grouped in four modules: 1. Time management 2. Physical demands 3. Mental/interpersonal demands 4. Output demands Respondents asked to rate the level of difficulty or ability to perform 25 specific job demands.	2 weeks	Not reported	No
Pelletier and Koopman, 2001, USA [32]	Stanford/American Health Association Presenteeism Scale (SAHAPS)	Measures the ability to concentrate on work among employees with health problems	General	No	42 items grouped in two modules: 1. Demographics 2. Presenteeism Respondents are asked to compare their usual performance and rate their performance using 5-point Likert scales and 0–100 % scales	1 month	Not specified but reported as suitable for hourly or salaried occupations	No
Koopman et al., 2002, USA [33]	Stanford Presenteeism Scale-6 (SPS-6)	Developed from the SPS-32 designed to assess the relationship between presenteeism and health problems.	General	No	Six-item questionnaire each designed to capture specific aspects related to presenteeism; 1. Cognitive 2. Emotional 3. Behavioural Respondents are asked to rate their health by stating the degree to which they agree on a scale of 1 to 5: 1 = strongly disagree, 5 = strongly agree.	1 month	Not reported	No
Gilworth et al., 2003, UK [34]	Work Instability Scale (WIS)	Develop a tool that can be used to indicate the level of risk of work disability	General	No	23-item questionnaire capturing information regarding the following: 1. Health 2. Work situation 3. Physical work factors 4. Hobbies The overall score indicates low, medium or at high risk of work disability. 0 = No problems at work, 4 = majority of job is unsuitable and individual is unlikely to cope.	Not Stated	Not reported	No
Goetzel et al., 2003, USA [35]	Work Productivity Short Inventory (WPSI)	Developed to gather information about absenteeism and presenteeism. Also gathers information about productivity loss if acting as the primary caregiver.	General	Yes	Questionnaire asks for the following: 1. Demographics 2. Employment status 3. Absenteeism 4. Presenteeism 5. Productivity loss associated with being a caregiver 6. The questionnaire asks about productivity loss associated with 15 health conditions. The respondent is asked to state number of days with the health condition, number of hours unproductive because of health condition and number of days missed from work. The same questions apply to those who are caregivers.	Three versions and vary only by recall period: 12 months 3 months 2 weeks	HCA	No
Kessler et al., 2003, USA [36]	The World Health Organisation Work Performance Questionnaire (HPQ)	Monetise the workplace costs of illness or cost savings of an intervention.	General	Yes	Three domains: 1. Work performance 2. Absenteeism 3. Job-related accidents Respondents asked to rate their overall work performance using a 0 to 10 scale: 0 = worst possible work performance, 10 = best possible work performance	Various; 1 week 4 weeks	Valuation of lost productive time is discussed. Authors do not recommend one method over another	No
Kumar et al., 2003, USA [37]	Health-Related Productivity Questionnaire-Diary (HRPQ-D)	Developed as a brief, self-administered instrument to be used within clinical trials and survey data collection.	General	Yes	Questionnaire asks for the following: 1. Premature retirement or reduction to part-time work 2. Absenteeism 3. Presenteeism Questionnaire designed in a diary format. Respondents are required to answer all questions for every day of the week. Some responses require the respondent to estimate their unproductive time other require respondent to choose from list of pre-specified %	1 day over 1 week	Not reported	No
Shikar et al., 2004, USA [38]	Health and Work Questionnaire (HWQ)	To assess various aspects of productivity without relying on only self-reported estimations	General	Yes	24-item questionnaire assessing the following 1. Work quality 2. Work quantity 3. Impatience 4. Concentration/focus 5. Work satisfaction 6. Non-work satisfaction Respondents are asked to rate their work performance using a 1 to 10 scale: 1 = worst and 10 = best	1 week	Not reported	No
Stewart et al., 2004, USA [39]	Work Health Interview (WHI)	To estimate the cost of illness of both absenteeism and presenteeism	General	Yes	Telephone interview. Six modules: 1. Informed consent 2. Employment status 3. Health conditions 4. Tasks and activities performed at work 5. Lost productive time (LPT): absenteeism and presenteeism 6. Demographics Respondents asked to choose the response that most applies, e.g. all of the time, some of the time, half of the time, none of the time. These responses are then converted into %	2 weeks	HCA	No
Turpin et al., 2004, USA [40]	Stanford Presenteeism Scale-13 (SPS-13)	Developed to assess presenteeism on (1) knowledge based and production jobs and (2) provide information on the health condition most likely to affect productivity	General	Yes	13-item questionnaire. Respondent states their primary health condition and is asked to base all answers given this health state. Presenteeism measured using the work impairment score which is the sum of responses to 10 Likert type questions. The final result is presented as a percentage of lost productivity.	4 weeks	Not reported	No
Ilmarinen et al., 2007, Finland [41]	Work Ability Index (WAI)	Assess work ability during health examinations and in workplace surveys	General	Yes	Seven-item questionnaire capturing information regarding the following: 1. Presenteeism 2. Health conditions 3. Absenteeism 4. Mental health Respondents rate their ability to work using various scales (1–10, 1–4 etc.). The index is calculated by summing the ratings given by the respondent.	Varies: 1 year 2 years Lifetime	Not reported	No
Osterhaus, Purcaru and Richard, 2009, USA [22]	Work Productivity Survey for Rheumatoid Arthritis (WPS-RA)	Estimate the productivity limitations associated with RA in paid jobs and unpaid work	MSD	Yes	Three-item questionnaire: 1. Employment status and occupation type 2. Absenteeism and presenteeism related to paid work 3. Absenteeism and presenteeism related to unpaid work Respondent is asked to rate the extent to which arthritis has interfered with their ability to work using a scale of 0–10: 0 = no interference to 10 = complete interference	1 month	Not reported	No
Prochaska et al., 2011, USA [42]	Well-Being Assessment for Productivity (WBA-P)	To create a measure of productivity based on well-being.	General		12 items assess reduced functioning related to personal and work well-being domains: 1. Personal: health, caring for others, financial, personal issues, depressed/stressed 2. Work: lack of resources, issues with co-workers, not enough time, issues with supervisors, lack of training, technical issues Respondents asked to choose response they most associate: not at all, some, a lot. A single number is estimated comprising of 11 items. The score ranges from 0 (not at all) to 100 (a lot for all 11 reasons).	4 weeks	Not reported	No
Zhang et al., 2012, Canada [43•]	The Valuation of Lost Productivity (VOLP)	Explicitly takes into account workplace characteristics necessary for valuing output loss resulting from input loss. The VOLP also used to calculate multipliers and compensation mechanisms.	General	Yes	Five modules: 1. Employment status 2. Absenteeism 3. Presenteeism 4. Unpaid work activity loss 5. Job and workplace characteristics Questionnaire also identifies information: 1. Team dynamics 2. Substitutability of work 3. Time sensitivity of output 4. Compensation 5. Availability of substitutes	7 days	HCA with multiplier Recommended method: Time multiplied by value of time (value of time = wage multiplied by relevant multiplier)	Yes
Boezeman et al., 2015, The Netherlands [44]	Composite Work Functioning Approach	Questionnaire that considers the relative importance of different aspects of work using weights	General	No	Questions based on WLQ and the Tilburg Psychological Contract Questionnaire. 1. Capacity to work 2. Quantity of work 3. Quality of work performance Respondents are asked to rate their ability to work using a 0 to 4 scale. Weights attached to different aspects of work functioning to reflect relative importance	Not stated	Not reported	No
Bouwmans et al., 2015, The Netherlands [45]	iMTA Productivity Cost Questionnaire (iPCQ)	To enhance the generalisability and comparability of outcomes for economic evaluations	General	Yes	Questions based on the short form HLQ and the PRODISQ. 18-item questionnaire. 1. Demographics 2. Absenteeism 3. Presenteeism 4. Unpaid work	4 weeks	HCA and FCA	No

^a^Listed in order of development

## Self-Reported Presenteeism Instruments: a History

The earliest identified measure of presenteeism, the Work Performance Scale (WPS), was designed by Jette et al. in 1986 [23]. The WPS asks the respondent to rate their ability to function physically, mentally and socially. The measure is simple and originally designed for clinical use. In 1993, Reilly et al. (1993) developed the Work Productivity Activity Index (WPAI) which differs substantially to the WPS. The WPAI asks the respondent to state the number of days missed from work and the number of days they found work difficult. The instrument also asks about productivity loss when doing unpaid work.

By the late 90’s and early 2000’s, presenteeism instruments were being designed to collect additional information regarding the contextual factors of an individual’s occupation. For example, the Occupational Role Performance Questionnaire (ORQ) developed by Kopec and Esdaile in 1998 [27] collects information about the individual’s job satisfaction, job security and the quality of the relationships they have with their colleagues. The Work Instability Scale (WIS) developed by Gilworth et al. in 2003 [34] asks questions about the respondent’s work situation and physical work factors.

In 2004, Stewart et al. [39] developed the Work Health Interview (WHI). The WHI is a telephone interview designed to collect information that can be used to estimate the cost of productivity loss. The interview introduces questions about the type of work tasks individuals are expected to complete as part of their job. The WHI is one of the first instruments that explicitly take into account how job characteristics may affect levels of presenteeism. In 2012, Zhang and colleagues developed the Valuation of Lost Productivity (VOLP) questionnaire [43•], a presenteeism instrument that explicitly takes into account how factors such as team dynamics, availability of perfect substitutes and time sensitivity of outputs either compensate or multiply levels of productivity loss caused by health conditions. Since 2015, presenteeism instruments, including the Composite Work Functioning Approach [44] and the iMTA Productivity Cost Questionnaire (iPCQ) [45], have been developed using questions from pre-existing measures including the Work Limitations Questionnaire (WLQ) [31], the Health Limitations Questionnaire (HLQ) [25] and the Productivity and Disease Questionnaire (PRODISQ) [46] rather than developing another completely new presenteeism instrument.

## Economic Theory Underpinning Presenteeism Instruments

Of the 24 studies that report the development of presenteeism instruments, only one study by Zhang et al. (2012) [43•] discussed how economic theory was used to underpin the design of their presenteeism instrument. They explain the economic rationale behind the development of their presenteeism instrument, the VOLP. The VOLP was designed to identify and measure productivity loss associated with various chronic health conditions and was validated using a sample of employees working with rheumatoid arthritis. Zhang et al. state that the concept of productivity is based on the theory of the production function, where output is a function of inputs including labour, capital and technology. Based on the economic theory of the production function, the authors define productivity loss due to ill health as the output loss associated with reduced labour input. Zhang et al. highlighted that no other existing presenteeism instrument captures both time input loss and information about workplace/job characteristics. The aim of the VOLP is to capture this information so that it can be used to measure productivity loss in terms of output loss associated with reduced labour input caused by ill health. Zhang et al. also critiqued the economics of valuing presenteeism using wage rates. Economic theory states that wages are assumed to be equal to the marginal productivity of workers. However, Zhang et al. argued that wages are often not an accurate reflection of the true value of productivity at the margin because of various other factors such as team production, availability of perfect substitutes and time sensitivity of outputs. Zhang et al. argue that these workplace and job characteristics need to be taken into account explicitly when attempting to measure productivity loss caused by ill health.

The remaining 23 studies stated that the motivation for the development of their presenteeism instruments was based on (1) the need to estimate the impact of presenteeism suitable for economic evaluations of healthcare and workplace interventions (18 studies: [22, 24, 25, 28–33, 35–42, 43•, 45]) and (2) the need to estimate individuals’ ability to function at work (5 studies: [23, 26, 27, 34, 44]). No formal theoretical framework of presenteeism from an economic or other relevant discipline, for example psychology, was used to underpin the methods used by the remaining 24 presenteeism instruments.

## Discussion

This systematic review has identified a substantial number of self-report presenteeism instruments, and one of these was specifically designed for use in an MSD-related condition (RA). With one exception, the development of the existing instruments was not underpinned by economic theory. Currently, the majority of self-reported instruments are atheoretical, which is problematic because the rationale, construct and development of the instruments cannot be linked. Zhang et al. [43•] was the only study that described how the economic theory of productivity was used to inform the design of the VOLP. The advantage of underpinning the design of the VOLP with economic theory of productivity is that it is clear what the rationale of the VOLP was, how it was designed and how it should be interpreted and used. Zhang et al. [43•] argue that most presenteeism instruments in the literature focus on measuring an individual’s labour input by measuring the time spent not working rather than the output lost from reduced labour. It is clear that in the absence of an economic theory of presenteeism, the interpretation of presenteeism from an economic viewpoint is contentious.

The absence of economic theory used to support the instrument to identify and measure presenteeism may have also contributed to the way in which researchers have approached the development of presenteeism instruments. The lack of a theoretical model for presenteeism means that researchers do not have a common framework from which to begin their research and develop their ideas. Therefore, as research into the measurement of presenteeism has grown, the instruments developed to quantify presenteeism have become more differentiated and more complex. The presenteeism instruments developed in the 1980s and 1990s are relatively simple where the respondent is asked to give information about their perceived level of absenteeism and presenteeism based on their health condition. In comparison, those presenteeism instruments developed in the late 2000s ask the respondent to consider a wide range of factors including job characteristics, team dynamics, time-sensitive output, job satisfaction, job security and relationships with colleagues, as well as the direct impact on presenteeism caused by their health condition. Ospina et al. [21•] and Noben et al. [47] recommended that the development of more self-reported presenteeism instruments is not needed and instead the literature should focus their effort on improving the ones that already exist. To some extent, this is happening where the two latest presenteeism instruments, the composite work functioning approach and the iPCQ, use questions from pre-existing presenteeism instruments. However, it is not yet clear which instruments are the most appropriate for measuring presenteeism in the context of health conditions in general, and MSD, specifically. The OMERACT group are currently working towards recommending which of the available measures is best used in the context of rheumatoid arthritis [48]. However, taking into account economic theory suggests the need to define the best measure in terms of clearly specifying the three constituent parts (identification, measurement, valuation) to quantify the impact of presenteeism.

## Limitations

The studies that were used to identify whether or not presenteeism instruments were developed using economic theory did not provide extensive detail. Many studies provided limited information that described how the presenteeism instrument was created. In an area where the quantification of a concept is subjective, such as presenteeism, it should be encouraged that researchers publish information about the conceptualisation and development of their presenteeism instrument. Such information would help inform the correct application and interpretation of their instrument, especially in the absence of applying an economic framework from which to underpin the instrument.

## Conclusions

This review has systematically identified all self-reported presenteeism instruments, providing a historical context and whether presenteeism instruments are underpinned by economic theory. With the exception of the VOLP, none of the instruments are explicitly underpinned by economic theory. One key area for further research is to take account of the need to understand how to identify, quantify and value the impact of presenteeism, while underpinning these stages with relevant economic theory for each constituent part of this process. Economic theory would aid the correct interpretation and application of the self-report presenteeism instrument and valuation approach. It is also vital that further development of presenteeism instruments are informed by robust empirical studies that take account of the context in which the final instrument will be used.

## Appendix. Electronic search strategies

OVID databases:

Medline (1946 to September week 3 2015),

Embase (1980 to week 40 2015),

CENTRAL (August 2015)

PsycINFO (1806 to September week 4 2015)

1. American Productivity Audit.tw.
2. “angina-related limitations at work question?aire”.tw.
3. endicott work productivity scale.tw.
4. “health and labo?r question?aire”.tw.
5. “health and performance question?aire”.tw.
6. “health and productivity question?aire”.tw.
7. “health and work question?aire”.tw.
8. health-related productivity question?aire*.tw.
9. “lam employment absence and productivity scale”.tw.
10. (migraine disability assessment adj2 (question?aire or survey or scale or score*)).tw.
11. MIDAS.tw. and migraine*.mp.
12. 10 or 11
13. (productiv* or presenteeism or absenteeism or work* or employ*).mp.
14. 12 and 13
15. “migraine work and productivity loss question?aire”.tw.
16. (osterhaus and (work* or productivity or presenteeism)).tw.
17. (osterhaus adj3 technique).tw.
18. “productivity and disease question?aire”.tw.
19. PRODISQ.tw.
20. (quantity adj2 quality adj (method or instrument)).tw.
21. (Stanford* adj5 Presenteeism Scale).tw.
22. “work and health interview”.tw.
23. work performance scale.tw.
24. “work productivity and activity impairment*”.tw.
25. WPAI*.tw.
26. (US National Health and Wellness Survey).tw. and (productiv* or presenteeism or absenteeism or work*).mp.
27. “health and work performance question?aire”.tw.
28. work productivity short inventory.tw.
29. wellness inventory.tw.
30. work role functioning question?aire.tw.
31. (or/1–9) or (or/14–30)
32. limit 31 to english language
33. (work productivity survey or WPS-RA).tw.
34. valuation of lost productivity.tw.
35. work limitations question?aire.tw.
36. (33 or 34) not 32
37. limit 36 to english language
38. 35 not 32
39. limit 38 to english language
40. 32 or 37
41. 32 or 37 or 39

ISI platform databases:

Web of Science (1900–November 6, 2015)

1. TS=“angina-related limitations at work questionnaire”
2. ((TS=“endicott work productivity scale”)) AND Language=(English)
3. (TS=(“health and labor questionnaire” OR “health and labour questionnaire”)) AND Language=(English)
4. ((TS=“health and productivity questionnaire”)) AND Language=(English)
5. ((TS=“health and work questionnaire”)) AND Language=(English)
6. ((TS=“health-related productivity questionnaire”)) AND Language=(English)
7. ((TS=“lam employment absence and productivity scale”)) AND Language=(English)
8. 8 ((TS=“migraine work and productivity loss questionnaire”)) AND Language=(English)
9. ((TS=“Stanford Presenteeism Scale”)) AND Language=(English)
10. ((TS=“health and work performance questionnaire”)) AND Language=(English)
11. ((TS=“work and health interview”)) AND Language=(English)
12. ((TS=“work performance scale”)) AND Language=(English)
13. ((TS=“work productivity short inventory”)) AND Language=(English)
14. ((TS=“American Productivity Audit”)) AND Language=(English)
15. ((TS=(osterhaus and (work or productivity)))) AND Language=(English)
16. TS=(“work productivity and activity impairment*”)
17. TS=WPAI*
18. TS=“wellness inventory”
19. TS=(“work productivity survey”)
20. TS=(“Work role functioning*” SAME limitations)
21. TS=(osterhaus SAME technique)
22. TS=(“productivity and disease questionnaire”)
23. (TS=(migraine disability assessment SAME (score* OR scale OR questionnaire OR survey))) AND Language=(English)
24. (TS=(MIDAS AND migraine)) AND Language=(English)
25. (TS=Osterhaus) AND Language=(English)
26. (#23 OR #24 OR #25) AND Language=(English)
27. (TS = (work* or productivity or performance or presenteeism or absenteeism or employ*)) AND Language=(English)
28. (#26 AND #27) AND Language=(English)
29. (TS=(“work role functioning questionnaire”)) AND Language=(English)
30. (TS=“health and performance questionnaire”) AND Language=(English)
31. TS=(“valuation of lost productivity”)
32. TS=(“work limitations questionnaire”)
33. (#1 OR #2 OR #3 OR #4 OR #5 OR #6 OR #7 OR #8 OR #9 OR #10 OR #11 OR #12 OR #13 OR #14 OR #15 OR #16 OR #17 OR #18 OR #19 OR #20 OR #21 OR #22 OR #28 OR #29 OR #30 OR #31 OR #32) AND Language=(English)

EBSCO databases:

CINAHL (1937–November 6, 2015)

Business Source Complete (1886–November 6, 2015)

**Table:**

Search ID#	Search terms
S28	S1 or S2 or S3 or S4 or S5 or S6 or S7 or S8 or S9 or S10 or S11 or S12 or S13 or S14 or S15 or S16 or S17 or S18 or S19 or S20 or S21 or S22 or S23 or S24 or S25
S25	“work limitations questionnaire”
S24	“valuation of lost productivity”
S23	“work productivity survey”
S22	“health and performance questionnaire”
S21	“productivity and disease questionnaire“
S20	osterhaus AND (technique or presenteeism or absenteeism or productivity or work* or employ*)
S19	“Work role functioning questionnaire”
S18	“wellness inventory”
S17	WPAI*
S16	“work productivity and activity impairment*”
S15	“American Productivity Audit”
S14	“work productivity short inventory”
S13	“work performance scale”
S12	“work and health interview”
S11	“health and work performance questionnaire”
S10	“Stanford Presenteeism Scale”
S9	“migraine work and productivity loss questionnaire”
S8	“lam employment absence and productivity scale”
S7	((“migraine disability assessment” n2 (score* or scale or survey or questionnaire)) OR (MIDAS AND migraine)) AND (work* OR productivity OR presenteeism OR absenteeism OR employ*)
S6	“health-related productivity questionnaire”
S5	“health and work questionnaire”
S4	“health and productivity questionnaire”
S3	(“health and labor questionnaire”) OR (“health and labour questionnaire”)
S2	“endicott work productivity scale”
S1	“angina-related limitations at work questionnaire”

Proquest databases:

ABI Inform (1970–November 6, 2015)

ALL ((“American Productivity Audit”) OR (“angina-related limitations at work questionnaire”) OR (“endicott work productivity scale”) OR (“health and labor questionnaire”) OR (“health and labour questionnaire”) OR (“health and productivity questionnaire”) OR (“health and work questionnaire”) OR (“health and performance questionnaire”) OR (“health-related productivity questionnaire”) OR (“lam employment absence and productivity scale”) OR (“migraine work and productivity loss questionnaire”) OR (“Stanford Presenteeism Scale”) OR (“work performance scale”) OR (“work and health interview”) OR (“health and work performance questionnaire”) OR (“productivity and disease questionnaire”) OR WPAI* OR (“work productivity and activity impairment*”) OR (“work productivity short inventory”) OR (“wellness inventory”) OR (“work role functioning questionnaire”) OR (“valuation of lost productivity”) OR (“work productivity survey”) OR (“work limitations questionnaire”) OR (((“migraine disability assessment questionnaire”) OR (MIDAS AND migraine) OR Osterhaus) AND (productivity or presenteeism or work* or employ*))
